# Identifying built environmental patterns using cluster analysis and GIS: Relationships with walking, cycling and body mass index in French adults

**DOI:** 10.1186/1479-5868-9-59

**Published:** 2012-05-23

**Authors:** Hélène Charreire, Christiane Weber, Basile Chaix, Paul Salze, Romain Casey, Arnaud Banos, Dominique Badariotti, Emmanuelle Kesse-Guyot, Serge Hercberg, Chantal Simon, Jean-Michel Oppert

**Affiliations:** 1 Lab-Urba, Urbanism Institute of Paris, University of Paris Est, Créteil, France; 2 UREN, INSERM U557/INRA U1125/CNAM/University of Paris 13/CRNH, Ile-de-France, Bobigny, France; 3 Image, Ville, Environnement, CNRS ERL730, University of Strasbourg, Strasbourg, France; 4 INSERM U707, University Pierre et Marie Curie-Paris 6, UMR-S 707, Paris, France; 5 CarMeN, INSERM U1060/INRA U1235/University of Lyon, CRNH Rhône-Alpes, Lyon, France; 6 Géographie-Cité, UMR 8504 CNRS, Paris, France; 7 Department of Nutrition, Pitié-Salpêtrière Hospital (AP-HP), University Pierre et Marie Curie-Paris 6, CRNH Ile-de-France, Paris, France

**Keywords:** Built environment, Urban form, Geographical Information Systems, Cluster analysis, Health-enhancing physical activity, Walking, Cycling, Body Mass Index

## Abstract

**Background:**

Socio-ecological models suggest that both individual and neighborhood characteristics contribute to facilitating health-enhancing behaviors such as physical activity. Few European studies have explored relationships between local built environmental characteristics, recreational walking and cycling and weight status in adults. The aim of this study was to identify built environmental patterns in a French urban context and to assess associations with recreational walking and cycling behaviors as performed by middle-aged adult residents.

**Methods:**

We used a two-step procedure based on cluster analysis to identify built environmental patterns in the region surrounding Paris, France, using measures derived from Geographic Information Systems databases on green spaces, proximity facilities (destinations) and cycle paths. Individual data were obtained from participants in the SU.VI.MAX cohort; 1,309 participants residing in the Ile-de-France in 2007 were included in this analysis. Associations between built environment patterns, leisure walking/cycling data (h/week) and measured weight status were assessed using multinomial logistic regression with adjustment for individual and neighborhood characteristics.

**Results:**

Based on accessibility to green spaces, proximity facilities and availability of cycle paths, seven built environmental patterns were identified. The geographic distribution of built environmental patterns in the Ile-de-France showed that a pattern characterized by poor spatial accessibility to green spaces and proximity facilities and an absence of cycle paths was found only in neighborhoods in the outer suburbs, whereas patterns characterized by better spatial accessibility to green spaces, proximity facilities and cycle paths were more evenly distributed across the region. Compared to the reference pattern (poor accessibility to green areas and facilities, absence of cycle paths), subjects residing in neighborhoods characterized by high accessibility to green areas and local facilities and by a high density of cycle paths were more likely to walk/cycle, after adjustment for individual and neighborhood sociodemographic characteristics (OR = 2.5 95%CI 1.4-4.6). Body mass index did not differ across patterns.

**Conclusions:**

Built environmental patterns were associated with walking and cycling among French adults. These analyses may be useful in determining urban and public health policies aimed at promoting a healthy lifestyle.

## Background

According to current socio-ecological models, both individual and neighborhood characteristics contribute to facilitating or limiting health-enhancing behaviors that may help to prevent chronic diseases [1-3]. In addition to social influences, the role of the built environment is being increasingly recognized in urban residential contexts [4-6]. As recently reviewed, environmental built characteristics such as the density of destinations, mixed land use and availability of sidewalks have been found to be associated with physical activity during leisure time and transport in different urban contexts [7]. There is also evidence that characteristics of the built environment related to physical activity are inversely associated with obesity in adults [8]. However, there is a need for additional evidence in each country and between countries.

Walking is the most frequent physical activity, as reported in surveys from North America, Australia and Europe; walking as well as cycling can be performed for both transport and recreation [9-11]. Walking and cycling represent priority targets for public health policies promoting physical activity, as they can be performed throughout the day at low cost. In addition, the promoting of these activities could be beneficial for both health and the environment [12,13]. A growing body of literature emphasizes the relationship between built environmental characteristics and walking [14,15]. The relationship between the built environment and cycling behavior in adults and, more specifically cycling for recreation and exercise, has been less thoroughly studied [12,16,17].

Proximity to potential destinations is one characteristic of the built environment that appears to be most consistently related to transportation and recreational walking [15]. In addition, the presence of cycle paths is an important environmental feature influencing cycling behavior [18,19]. Relationships between green spaces and recreational walking, physical activity and weight status have also been reported [20-23]. Importantly, walking and cycling may be specifically associated with given combinations or patterns of characteristics of the built environment [24]. Using this type of approach, Riva et al. [25] used cluster analysis of data on population density, land use mix and accessibility to proximity services at a census tract level on the island of Montréal; they obtained seven types of active living potential environments.

Cities represent complex systems [26], and urban forms and their overall organization vary widely between Europe, Northern America and Australia [27,28]. This indicates the need for data from different countries and settings [29]. Up until now, European analyses mainly focused on relationships between perceived environmental dimensions and physical activity behavior. Positive associations were reported in Belgium [30] and Portugal [31]. Fewer European studies have investigated the link between objective environmental measurements and physical activity behavior [29]. For example, a Belgian study reported positive associations between high walkabability, walking/cycling for transport and recreational walking [29]. To our knowledge, no European studies have investigated the possible relationship between built environmental patterns and walking/cycling for exercise and recreation.

The first aim of the present study was to identify built environmental patterns in a French urban context using data on green spaces, cycle paths and proximity facilities in and around Paris. An additional aim was to assess associations between these environmental patterns, body mass index (BMI) and recreational walking and cycling behaviors, as performed by middle-aged adult residents.

## Methods

### Geographic information systems (GIS) environmental characteristics

**Figure 1:**
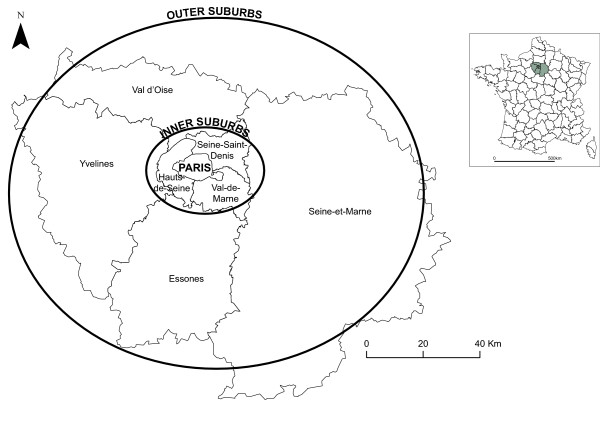
Urban ring model of Paris and seven surrounding departments (Ile-de-France region): Paris, inner suburbs and outer suburbs.

The study area included the city of Paris and seven surrounding departments defined administratively as the “Ile-de-France”. The overall area covers 12,011 km² and has more than 11.7 millions inhabitants according to the 2010 French census (see the French National Institute of Statistics and Economic Studies (INSEE) website at http://www.insee.fr). The Ile-de-France region is traditionally divided into “ring spatial division” comprising three areas: Paris city, inner suburbs and outer suburbs, as represented in Figure 1.

Objective built environment variables were obtained using GIS for all census units IRIS (n = 5,261) in the region. French IRIS areas (http://www.insee.fr) represent neighborhoods on a scale comparable to a census block group in the US. This is the smallest unit for which population census data are available in France. IRIS areas include on average 2,000 inhabitants and are homogeneous in terms of housing and socioeconomic conditions (http://www.insee.fr).

For each neighborhood, we assessed three geographic variables: spatial accessibility to green spaces (urban parks, public open areas, natural areas and green paths), spatial accessibility to proximity facilities and the availability of cycle paths. Green space data were obtained from databases provided by the Paris Region Urban Planning & Development Agency (IAU, Ile-de-France) [32]. For each green space, a catchment area (buffer) was calculated according to both size and form of the green space: linear (such as a green path) or surface (such as an urban park), and to geographical limitations (e.g. transport network or river). For green paths, the buffer was 300 m wide for lines which were 300–1,000 m long, 600 m wide for lines of 1,000–5,000 m and 1,200 m wide for those over 5,000 m. For green surfaces, the buffer was 300 m for green spaces between 1–10 hectares (ha), 600 m for green spaces between 10–30 ha and, finally, 1,200 m for those over 30 ha [32]. The variable representing spatial accessibility to green spaces was defined as the percent of green spaces in each neighborhood included in at least one catchment area.

For cycle paths, data were obtained from databases also provided by the Paris Region Urban Planning & Development Agency (IAU Ile-de-France). We used the length of cycle paths; the variable used in analyses showing the availability of cycle paths was the distance in kilometers for each neighborhood.

Data on geographic distribution of proximity facilities (banks, bakeries, post offices, drugstores and bookstores) were obtained from the INSEE facilities census (http://www.insee.fr). Spatial accessibility to selected local facilities was estimated by a potential accessibility model [33,34] according to the number of proximity facilities (banks, bakeries, post offices, drugstores and bookstores) in built-up areas within one kilometer Euclidean distance computed from the centroïd (geographical center) [35] of the IRIS administrative unit. Briefly, this spatial accessibility model defined an intensity index of the possible destinations. In the model, these destinations are weighted according to an inverse function of distance. In addition, spatial smoothing was applied to remove artificial barriers defined by administrative borders and to obtain a more realistic estimate of accessibility levels. As previously described [34], the potential accessibility model was implemented in the XLISP-STAT programming environment, and ArcGIS 9.3 (ESRI, Redlands, CA) was used to produce an objective measure of the built environment.

Finally, the spatial accessibility indices for proximity facilities and green catchment areas were categorized into quartiles. The top and bottom quartiles represented high accessibility and low accessibility, respectively. The indicator of availability of cycle paths was also divided into four categories: the first one represented neighborhoods without cycle paths, and the three other categories were defined using tertiles cut-offs.

### Built environmental patterns

A classification procedure based on the successive use of two multivariate statistical methods was used to identify built environmental patterns using GIS objective data. This procedure employed Multiple Correspondence Analysis (MCA) followed by cluster analysis based on hierarchical ascendant classification [36,37].

In a first step, MCA was used to explore interrelationships between multiple dependent variables. This method enables determining the dimensions that provide the most information about relationships between variables [38]. The number of dimensions retained was determined according to the following criteria: eigenvalue > 1, screen test (plot of the total variance related to each dimension) and interpretability of MCA [36,39]. In our study, groups of spatial accessibility to green areas and proximity facilities and availability of cycle paths defined for each neighborhood were included in the model and treated simultaneously.

In a second step, to identify neighborhood patterns, cluster analysis was performed only with dimensions retained through the MCA procedure (in such a way that a given area belonged to one, and only one, pattern) [36,40]. In the present study, patterns provided by cluster analysis were based on similarities in GIS environmental variables (i.e. green areas, proximity services, cycle paths). Ward’s method was used to define patterns [36,41] and a statistical criterion (“test-value”) was used to interpret patterns. As described elsewhere [9,39], we assessed differences between each category of each variable, characterizing the cluster and the relevant category in the overall sample according to a statistical criterion based on a “test-value”. In cluster analysis, this test value is interpreted as a criterion that enables classifying categories of the variable by order of importance, thereby facilitating interpretation of results. A positive test value higher than two means that the category of the variable is significantly overrepresented in the cluster compared to the overall sample [9,39]. Note that this procedure allows two categories of the same variable to be overrepresented in the same cluster. MCA and cluster analyses were conducted using SPAD software (Coheris, version 7.3).

### Socio-environmental covariables

The median annual income area data in 2006 from the Tax Income Files (http://www.insee.fr) was used as an indicator of the environmental socio-economic position. Income data from all neighborhoods were categorized into quartiles. The top and bottom quartiles represented high and low income levels, respectively.

### Individual data

Individual data were obtained from participants in the SU.VI.MAX cohort. The SUpplémentation en VItamines et Minéraux AntioXydants (SU.VI.MAX) study was initially a randomized, double-blind, placebo-controlled, primary prevention trial designed to evaluate the impact of daily antioxidant supplementation at nutritional doses on the incidence of ischemic heart disease and cancer [42,43]. A total of 5,056 men aged 45–60 years and 7,679 women aged 35–60 years from throughout France were included between October 1994 and June 1995, with a planned follow-up of 8 years. Details on recruitment, study design and main results of the study have been reported previously [42,43]. All subjects gave informed written consent for the study, which was approved by the Ethical Committee for Studies with Human Subjects at the Paris-Cochin Hospital (CCPPRB N^o^706) and the Commission Nationale Informatique et Liberté (CNIL N^o^334641). In 2007–2009, 6,850 participants in the cohort whose agreement was obtained were enrolled in the SU.VI.MAX 2 study, an observational prospective cohort study designed to investigate the relationship between nutrition and health status in an ageing population [44]. The SU.VI.MAX 2 study was approved by the local ethical committee (CCP N° 2364) and the Commission Nationale de l'Informatique et des Libertés (CNIL N° 907094). Signed informed consent was obtained from each participant.

### Individual level of walking and cycling, weight status and sociodemographic characteristics

For the present analysis, in order to obtain a similar age range in both sexes, the sample was further restricted to subjects aged 45 or older in 1998. We selected subjects who provided data on leisure time physical activity in 2007 (i.e. the most recent year during which a detailed leisure physical activity questionnaire was sent to the entire cohort) and who resided in the Ile-de-France region in 2007. We also excluded subjects who had been confined to bed for more than 1 month during the period covered by the physical activity questionnaire. Analyses in the present report were thus based on data from 603 men and 706 women.

Data on time spent walking and cycling were obtained using a self-administered French version of the Modifiable Activity Questionnaire (MAQ) [45]. The MAQ has been developed by Kriska et al. to investigate relationships between habitual level of physical activity and diabetes [46]. Criterion validity of the MAQ was assessed against doubly-labelled water [47] and the questionnaire has been described in detail elsewhere [45-47]. Briefly, subjects were asked to report all leisure physical activities performed at least 10 times for 10 min per session over the past 12 months. Then, detailed information was collected about the type of physical activity (eg. walking, biking) as well as the frequency and duration of each physical activity reported. The assessment of walking and biking was based on the total number of hours per week for each subject. We calculated the total median duration for walking and biking (min/week) among subjects who had reported performing at least one of these activities and we defined three categories for performing walking and cycling: none, less than the median and equal to or above the median value.

Body weight was measured using an electronic scale (Seca, Hamburg, Germany) with the subject wearing indoor clothes and no shoes [48,49]. Height was measured under the same conditions to the nearest 0.5 cm using a wall-mounted stadiometer. Anthropometric data were collected at the inclusion visit in the SU.VI.MAX 2 study (2007–2009). BMI was calculated as body weight divided by height squared (kg/m²).

Individual sociodemographic data were obtained through questionnaires at study entry (sex, age, education level). Age in 2007 was divided into three groups (50–59, 60–69 and ≥70 years). Level of education was coded into three categories according to the highest certification obtained (primary school, high school, university or equivalent).

### Analysis of relationships with individual behaviors

The associations between built environmental patterns and walking and cycling time were analyzed with multinomial regression models. This analysis was conducted using the category of subjects who did not report walking or cycling as the reference category for the dependent variable. Using covariance analysis, we assessed the associations between built environmental patterns and BMI. Regression and covariance analyses were performed using SAS software (SAS, version 9.3, SAS Institute Inc., Cary, NC, USA). Based on previous literature and on exploratory analyses, adjustment was performed for individual potential confounding factors including age, gender and education level, and for median annual income of neighborhood as an environmental socioeconomic factor.

## Results

### Identification and geographic distribution of built environmental patterns

**Figure 2:**
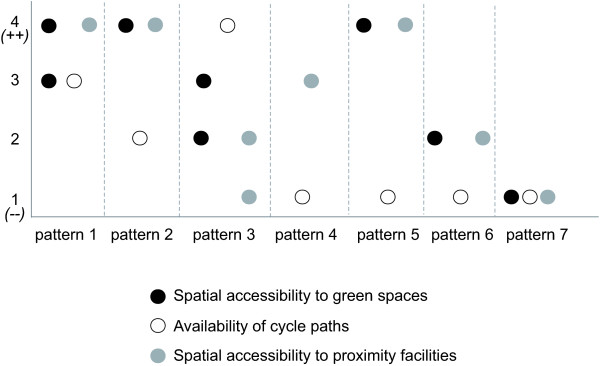
**Characteristics of the seven built environmental patterns according to categories of spatial accessibility to green spaces, proximity facilities and availability of cycle paths.** On the y axis are represented the 4 categories used for each variable. For green spaces and proximity facilities, the top and bottom quartiles represented high (++) and low accessibility (−−). For cycle paths, (−−) represented neighborhoods without cycle paths and the three other categories were defined using tertiles. Dots indicate those categories overrepresented in each cluster compared to the overall sample. For example, for Pattern 1, both quartiles 3 and 4 for spatial accessibility to green spaces were overrepresented.

The first two neighborhood dimensions generated by MCA that accounted for 31.7% of total initial variance in environmental characteristics were used to conduct cluster analysis. Cluster analysis identified seven distinct neighborhood clusters. Figure 2 illustrates the characteristics of each cluster according to categories of the three built environmental variables (green areas, cycle paths and proximity facilities). Each dot in a given cluster represents a category of a built environmental variable that is significantly overrepresented in the cluster compared to the overall sample.

Mapping of the seven patterns enabled us to highlight the geographic location of each pattern across neighborhoods in the region (Table 1). In Pattern 1, overrepresented categories included high spatial accessibility to local services, high spatial accessibility to green spaces (quartiles 3 and 4) and high availability of cycle paths. Neighborhoods characterized by this pattern (n = 794, 15.1% of neighborhoods) were found in the city of Paris, but were also localized at about the same proportions in the suburbs. Pattern 2 was characterized by high spatial accessibility to local services and green spaces, but by low availability of cycle paths. It was also localized both in Paris and the suburbs. Pattern 3 (n = 794, 15.1%) was characterized by low spatial accessibility to local services; in contrast, the spatial accessibility to green spaces and availability of cycle paths were high. We mainly observed this pattern in the outer suburbs (n = 494, 62.2%), but also in urban residential areas at the borders of Paris (n = 103, 12.9%) and in the inner suburbs (n = 197, 24.8%). Pattern 4 (n = 755, 14.4%) reflected neighborhoods characterized by high spatial accessibility to local services, by the absence of cycle paths, and it was localized in the suburban areas (more in the inner than in the outer suburbs). Pattern 5 (n = 629, 12.0%) reflected a group of neighborhoods characterized by high access to green spaces and proximity facilities and by an absence of cycle paths. Pattern 5 was mainly found in Paris and the inner suburbs. Pattern 6 (n = 663, 12.6%) was characterized by an absence of cycle paths and by medium spatial accessibility to proximity facilities and green spaces, and was localized in both types of suburbs. Pattern 7 (n = 833, 15.8%) was mainly localized at the borders of the outer suburbs in neighborhoods characterized by low spatial accessibility to green spaces and proximity facilities and by an absence of cycle paths.

**Table 1:** Geographic distribution of built environmental patterns in the Ile-de-France region

	**Pattern 1**		**Pattern 2**		**Pattern 3**		**Pattern 4**		**Pattern 5**		**Pattern 6**		**Pattern 7**	
	**n**	**%**	**n**	**%**	**n**	**%**	**n**	**%**	**n**	**%**	**n**	**%**	**n**	**%**
Paris	229	28.8	265	33.4	103	12.9	1	0.1	394	62.6	0	0	0	0
Inner suburbs	245	30.9	302	38.1	197	24.8	512	67.8	209	33.2	252	38.0	40	4.8
Outer suburbs	320	40.3	226	28.5	494	62.2	242	32.1	26	4.1	411	62.0	793	95.2
Total	794	15.1	793	15.1	794	15.1	755	14.3	629	12.0	663	12.6	833	15.8

### Individual characteristics

Characteristics of subjects, including sociodemographic characteristics, walking and cycling behaviors and BMI, are shown in Table 2. Mean ± s.d. age was over 60 in both genders. About two-thirds of men and half of the women were retired in 2007. For both genders, more than 60% of subjects performed recreational walking. In walking and cycling duration categories, 25.9% of subjects (339) did no walking or cycling.

**Table 2:** Characteristics of study subjects

	**Men (n = 603)**		**Women (n = 706)**	
	**N**	**%**	**N**	**%**
Age				
Age (year) ^1^	64.9 ±4.6		62.9 ±5.3	
50-59 years	82	13.6	198	28.1
60-69 years	389	64.5	408	57.8
≥70 years	132	21.9	100	14.2
Education level				
Primary school	132	21.8	125	17.7
High school	193	32.0	263	37.3
University or equivalent	267	44.4	296	41.9
Missing value	11	1.8	22	3.1
Working status				
Not retired	153	25.4	271	38.4
Retired	415	68.8	361	51.1
Missing value	35	5.8	74	10.5
Physical activity performed				
Walking	425	70.5	453	64.2
Cycling	178	29.5	102	14.4
No walking or cycling	0	0.0	151	21.4
Median total duration h/week (Q1-Q3) Aligment with Walking/Cycling	1.15 (0–3.5)		1.1 (0.1-3.1)	
Body mass index (kg/m²)^1^	26.3 ±3.4		24.6 ±4.3	

^1^Mean ± standard deviation.

### Relationships with built environmental patterns

**Figure 3:**
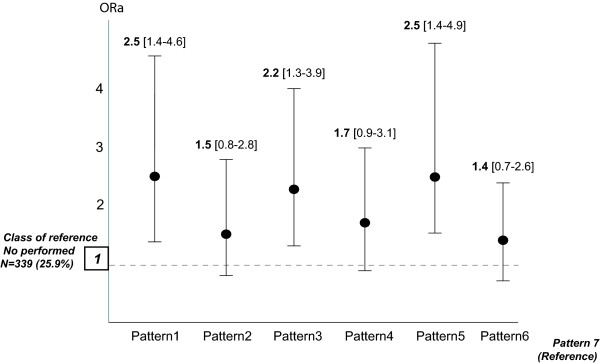
**Adjusted**^**a**^**odds ratios (ORa) for the likelihood of walking and cycling over weekly duration categories (n = 487, 37.2%) by built environmental patterns**^**b**^**.**^a^Adjusted for individual characteristics (age, education level) and socio-economic environmental characteristics (median income) ^b^Built environment patterns (1 to 7) are those described on Figure 2.

In multinomial regression models, pattern 7 was used as the reference pattern. Associations between built environmental patterns and walking and cycling categories are shown in Figure 3. After adjustment for individual characteristics (age and education level) and median neighborhood income, the likelihood of walking and cycling over the median weekly duration was increased in pattern 1 compared with the reference pattern (pattern 7) (OR = 2.5 95%CI 1.4-4.6). The likelihood of walking and cycling also increased for both patterns 3 (OR = 2.2 CI95% 1.3-3.9) and 5 (OR = 2.5 CI95% 1.4-4.9) compared with the reference pattern. There were no significant associations between built environmental patterns and the category of subject who had reported walking and cycling less frequently than the median weekly duration. There was no significant difference in BMI across patterns (data not shown).

## Discussion

The first aim of this study was to identify built environmental patterns in a European urban context. To approach the complexity of urban forms in European settings, we used a methodology enabling us to analyze the co-occurrence of several important characteristics of the built environment. Using GIS datasets of urban characteristics and a combination of MCA and cluster analysis, we identified seven built environmental patterns in the region around Paris, France. A pattern characterized by low spatial accessibility to green spaces and proximity facilities and an absence of cycle path was found only in neighborhoods in the outer suburbs, whereas patterns characterized by higher spatial accessibility to green spaces and proximity facilities and the presence of cycle paths were more evenly distributed across the region. In addition, we found an increased likelihood of walking and cycling in subjects residing in neighborhoods characterized by high accessibility to green spaces and proximity facilities compared to those living in neighborhoods with low spatial accessibility to green spaces and proximity facilities and without cycle paths, after adjusting for individual sociodemographic characteristics (age, gender and education level) and median neighborhood income.

One strength of the present study lies in our analysis of built environmental patterns that take into account specificities of the physical environment in urban contexts. Examination of spatial distribution of the seven patterns and analyses of the relation between these patterns and walking and cycling behaviors reveal the complexity of the urban built environment in this study region (Ile-de-France), beyond a simple division between the city center, the inner and the outer suburbs. In the current investigation, we were specifically interested in pedestrian and cycling-related built environments based on three variables: green spaces, cycle paths and proximity to facilities. Previous research suggested the importance of built environment attributes facilitating walking, such as high residential density, street connectivity and land use mix [24,50-52]. In the present study, information on residential density was not included in the analyses of environmental patterns because of strong correlations observed between population density (using built-up area density) and spatial accessibility to proximity services (Spearman rank correlation = 0.73 p < 0.0001). In addition, information on the presence of sidewalks on both sides of the street (a component of walkability) was not found relevant to the situation in France. We estimated the availability of sidewalks (length of sidewalks in kilometers in each neighborhood in Paris) and the density of road network (excluding large roads and highways) in each neighborhood of the study region; however, the variability of these characteristics was not significant (data not shown). In French suburbs, there are almost always sidewalks alongside the streets, and these may include cycle paths. Indeed, our patterns with high availability of cycle paths were localized both within Paris and in the suburbs (inner and outer).

To categorize neighborhoods into homogeneous patterns according to built environmental characteristics, we used a combination of MCA and hierarchical cluster analysis. This approach has been used in previous studies to define social and demographic neighborhood patterns and to assess individual health outcomes according to these social patterns [53]. As described by Escofié et al. [36], MCA was used as a preliminary step for classification due to its role as a filter for eliminating non-relevant dimensions that might be assimilated with ‘statistical noise’. In a second step, most significant factorial dimensions obtained by MCA were included in cluster analysis to generate groups of neighborhoods with similar built environmental characteristics. Integration of these two methods enables analyses based on the most significant interrelations between categories of built environmental variables [54] and might better explain the diversity and natural grouping of characteristics, thereby overcoming limitations due to use of either factor or cluster analysis [53].

An important feature of our study was that we were able to assess the relationships between built environmental patterns and walking and cycling behaviors in a sample of middle-aged adult residents. We found that patterns 1, 3 and 5 were significantly associated with the likelihood of walking and cycling over the median weekly duration. In order to generate hypotheses concerning these relationships with built environmental patterns, we assessed the geographic distribution of the seven neighborhood patterns. The principal difference between patterns 1 and 3, both localized across the three zones of the region, was in the availability of cycle paths, lower in pattern 3 than in the reference pattern. There is evidence that the presence of cycle paths is important in creating an active living local environment. In New Orleans, an increase in the average number of riders (both adults and children) per day was observed after the first on-street bike lane was painted on the streets of the city in 2007 [18]. However, in our study, increased likelihood of walking and cycling was also found for pattern 5, characterized by an absence of cycle paths. Depending on the urban context, this relationship may be due to other aspects of the built environment that may influence walking and cycling, such as specific equipment in the green spaces [21,55,56] or the amount of traffic [57].

In contrast to positive relationships with walking and cycling of some built environment patterns, no significant association was found with BMI. This is consistent with findings from a recent systematic review [5] which suggested that some built environmental features were associated with an increased level of physical activity, especially walking, but not with BMI. In addition, in another recent review [20] the relationship between green space and weight was found inconsistent. As emphasized by the UK Foresight Report [58], excess weight and obesity represent highly complex systems shaped by multiple interdependent factors acting throughout the lifespan. Therefore, to be able to demonstrate a direct and simple relationship between built environment patterning and weight status would be unexpected. Especially, the cross-sectional design of available studies cannot take into account potential time-lags between exposure to built environment and body weight change [20], pointing to the importance of longitudinal studies.

In the present study, walking and cycling data were available only for recreational physical activity. It should be noted, however, that more than 50% of study subjects (68.6% in men and 51.1% in women) were retired. Therefore, information on commuting activities may not be relevant. In a recent systematic review focusing on older adults (over 65 years), Van Cauwenberg et al. [59]. pointed out that, while non-significant associations between walkability indices and recreational walking were found in the US and Australia a significant positive association was found in Belgium. In a European urban context, Van Dyck et al. assessed the link between walking and cycling (transport and recreation) and the level of walkability in an urban city (Ghent) in Belgium [29]. That study showed that a high level of walkability was positively associated with a high level of walking both during transport and recreation.

### Limitations

Several limitations of our study need to be pointed out. The design was cross-sectional and thus we could not establish causal relationships between built environmental patterns and walking and cycling behaviors. Nor can we rule out a bias related to residential neighborhood self-selection. Neighborhood selection may be determined by numerous factors based on financial constraints, availability of equipment, transportation infrastructures and lifestyle [60,61]. In our study, subjects were volunteers in a nutritional intervention study [42,43]. Although characteristics of the participants of the SU.VI.MAX study were found to be close to those of the national population according to socioeconomic status and to the distribution of major risk factors for cardiovascular disease and cancers [42,43], these subjects may have had healthier lifestyles.

Another limitation was that measurements of walking and cycling were derived from self-report and, as noted in previous studies [62,63], this may be a source of potential misclassification. In general, it is known that physical activity (especially information concerning duration and frequency) tends to be overreported and sedentary behavior is underestimated [62]. In addition, although our patterns were based on analyses of objective GIS built environmental data, we lacked information on environmental variables which may promote or limit walking and cycling behaviors, such as road traffic and safety [64], and specific characteristics of green spaces such as access points and the presence of equipment [21,55,56]. Another limitation is related to geographic scale and area arrangement. Results are likely to vary with the size and the arrangement of areas, with larger administrative units being more heterogeneous, which may occur when the same range of data, calculated at various spatial levels, produces different results [65,66]. To limit a potential bias related to the relationship between size and location, the variable used for measuring spatial accessibility to facilities was defined according to the built-up area of each IRIS.

Finally, our study territory was limited to one European city (Paris and suburbs); further studies are needed to determine whether they can be generalized to other European urban settings.

## Conclusions

Based on accessibility to green spaces and proximity facilities and the availability of cycle paths, we identified specific local built environmental patterns in a French region. Using individual physical activity data, we were able to show that subjects living in neighborhoods characterized by high accessibility to green spaces and proximity facilities and high availability of cycle paths did significantly more walking and cycling than those living in neighborhoods with low accessibility and availability. No significant relationship was found for overall corpulence, as assessed by the BMI. These findings emphasize the complexity of urban forms at regional and local levels, and suggest the need to provide culture-specific approaches to characterizing neighborhood contexts in relation to healthy behaviors. Physical inactivity is recognized as a major risk factor for ill health and the onset of non-communicable diseases [67]. In this context, our analyses may be useful for influencing urban and public health policies aimed at promoting healthy lifestyles in urban settings.

## Abbreviations

GIS, Geographical Information Systems; BMI, Body Mass Index; INSEE, French National Institute of Statistics and Economic Studies; IRIS, “Ilôts Regroupés pour l’Information Statistique”; IAU, Paris Region Urban Planning & Development Agency; MCA, Multiple Correspondence Analysis; MAQ, Modifiable Activity Questionnaire.

## Competing interests

The authors declare that they have no competing interests.

## Authors’ contributions

HC designed the study, performed statistical analyses, interpreted results and drafted the manuscript. CW, BC, PS, RC, AB, DB, EKG, SH and CS participated in interpreting results, editing the manuscript and critically revising it. JMO supervised the design, analysis and interpretation of data, as well as writing of the manuscript. SH is a coordinator of the SU.VI.MAX study. All authors read and approved the final manuscript.
